# Keeping patients safe: a practical guide

**Published:** 2015

**Authors:** Janet Marsden

**Affiliations:** Nurse Advisor: Community Eye Health Journal, London, UK. Email: J.Marsden@mmu.ac.uk

**Figure:**
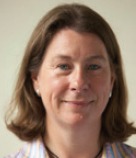
Janet Marsden

Patient safety is the prevention of avoidable errors and the harm they cause to patients; it is the foundation of good patient care. Achieving safety in patient care is part of achieving quality patient care.

When we think about patient safety, we normally think about the physical safety of the patient in terms things like prevention of cross-infection and safe site surgery and, indeed, we will consider those here. We also need to think about the non-physical aspects of patient safety – which are sometimes more difficult to deal with – so that we can achieve care that is **patient centred.** This means providing care that is respectful of and responsive to patient needs, beliefs and preferences; in other words, working **with** patients rather than doing things **to** them. This article will therefore also consider informed consent to treatment and some of the principles underlying working with patients in a patient-centred way.

## Preventing harm and infection control

Preventing infection and cross-infection is important to protect both patients and health care professionals. It is very easy to transfer infection-causing organisms (e.g. viruses and bacteria) from one place to another and therefore from one person to another. It is important in day-to-day life, but even more important in the hospital setting where patients may have reduced immunity or wounds, making them vulnerable to infection.

## Hand washing

Hand washing is one of the most important actions we can take to prevent the spread of infections and therefore prevent loss of health, or even death. Hand washing will also protect health workers from infection and save money by reducing the need for expensive treatments once infection has occurred. When practiced routinely, it also provides a good example to patients, relatives and others in the eye team.

### Before you hand wash

Remember that organisms can sit under your watch, your rings and your nail polish. This can make any hand washing ineffective. You should remove your watch, any rings with stones in (because of the dirt that can sit around the stone) and you really should not wear nail polish! Bacteria and dirt can sit under the end of a long nail so it is important to have short nails when you work in health care, and especially in ophthalmology where we often have our fingers very close to the eye.

**Figure 1.**
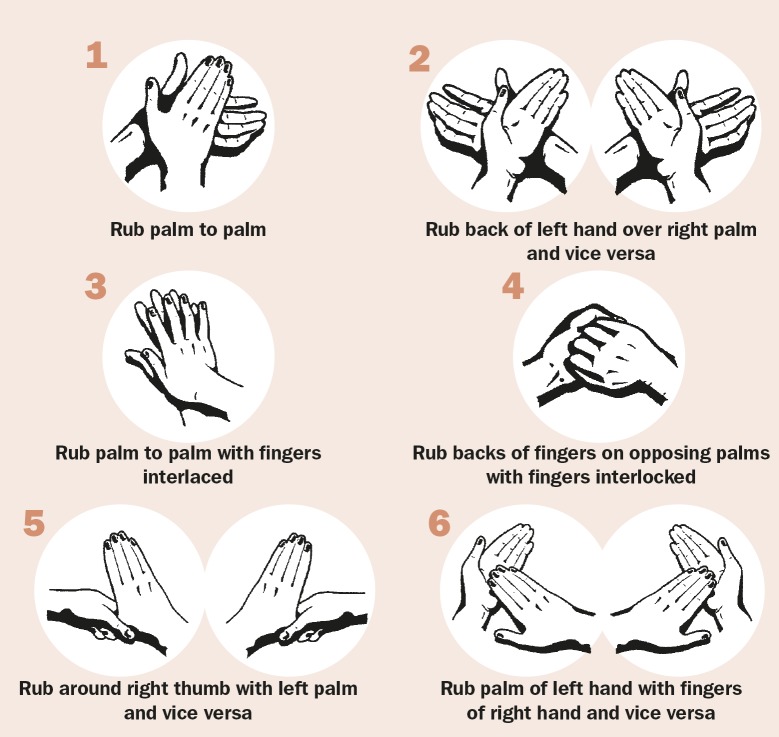
Six steps to handwashing

Scratches and wounds on your hand can harbour infection and can also become infected. They should be covered.

Once you have no jewellery, no watch, no nail polish, no false nails, no long nails, no cracked skin or open wounds (including cuts or cracked skin), **you are ready to wash your hands.**

Before you start to wash, work out why you need to wash your hands. Are you handwashing:

- at home, in between tasks?
- at work, while caring for patients?
- in surgery?

### Everyday hand washing

You should do this before touching food, after using the toilet, after gardening or playing with pets, or doing anything that is dirty. It can be a 30–40-second wash with soap and clean water, with your hands dried on a clean towel.

### Hand washing in a care setting

This should take place before and after caring for a patient, after using the toilet, before and after food, and after dealing with any dirty or contaminated material. It should be a 30–40-second wash, as at home: cleaning the whole hand and wrist. This can be carried out with hospital approved liquid soap and water (solid soap can harbour organisms) or with antimicrobial gel or foam (hand sanitiser). You should dry your hands with a clean disposable paper towel and make sure that they are dry before you do your next task. The type of soap used at home is generally not adequate for use in the hospital but if you have nothing else, it is certainly better than nothing.

If you are using hand sanitiser, rub it into all the surfaces of your hands and wrists, as you would when washing with water, until it has dried.

### Surgical scrub

This is a systematic and much longer hand wash and scrub using an antiseptic washing solution before surgery.

In the hospital or care setting, the soap or solution to be used should be placed next to where you should use it, but do check if you are not sure. Dispensers and taps usually have long handles which can be operated by the elbow, so that scrubbed hands remain absolutely clean.

## Hand hygiene

The World Health Organization's (WHO) **5 Stages for Hand Hygiene**^1^ is applicable everywhere and states that you should wash your hands:

1. Before touching a patient.
2. Before clean/aseptic procedures.
3. After body fluid exposure/risk.
4. After touching a patient.
5. After touching patient surroundings.

Concerning hand washing, all health workers have the responsibility to:

- Practice safe hand washing techniques.
- Be pro-active: lead by example and encourage others to do the same.
- Educate patients and colleagues.

## Clean patients

Before surgery, the patient needs to be clean. There is little point in the nurses and health care workers making sure that they, the theatre and the instruments are clean if the patient is bringing contaminants into the theatre. It is risky for them and it is risky for other patients in the area. Depending on local circumstances, the patient may wash at home or in the hospital but they will need information before their operation so that they know about this. When the patient is in hospital, it is good practice to provide a culturally appropriate environment where they are able to wash daily, and to encourage hand washing before food and after toileting. It helps to maintain good hygiene standards at the health facility and sets a good example which they can then take home with them.

**World Health Organization's Safe Site Surgery**

It is important that the right patient gets the right operation to the right part of their body with the right methods at the right time.

It can be a very damaging for all concerned if this does not happen. In order to help this process, the World Health Organization's (WHO) Safe Site Surgery Guidelines^2^ was developed.

The guidelines suggest that checks are done at three points in time: before anaesthesia (sign in), before incision (time out), and before the patient leaves the operating room (sign out). These are the points in time when everybody involved stops what they are doing to focus on the safety of the patient.

The checklist should be completed by all members of the medical team, including the surgeon and anaesthetist. These checklists have now been incorporated into hospitals around the world.

Figure 1 gives the generic WHO surgical safety checklist which can be adapted to suit the hospital or procedure. For example, the United Kingdom has its own cataract surgery safety checklist, which is available online.^3^

Before starting the operation, all members of the team within a particular clinical area must check that the patient's details in the chart correspond with the patient in front of them and that they have all the equipment, stock, medications and instruments required (the medication and stock must be in-date) and that any issues for each department have been raised, discussed and followed up as needed.

**Figure 2.**
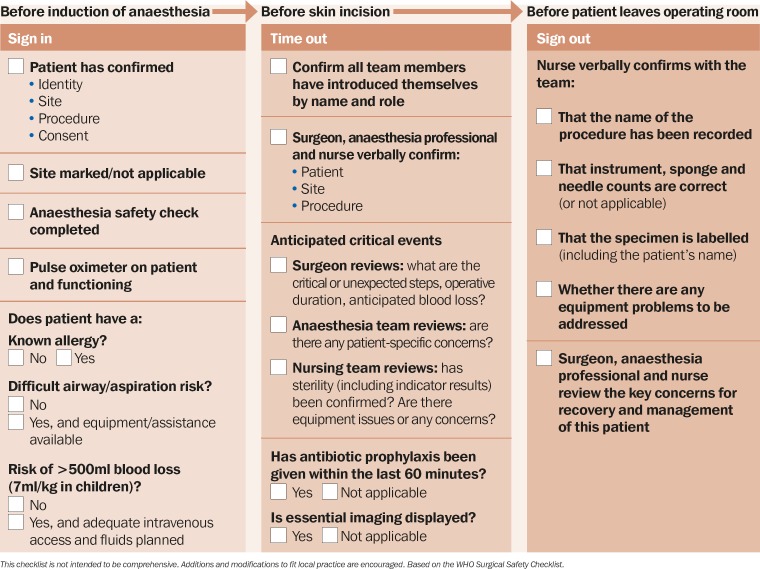
Surgical safety checklist

## Waste management

Make sure a policy on waste management is familiar to all staff. This includes ensuring that contaminated waste is disposed of correctly. Gloves must be worn when handling waste and hand washing is a must after touching contaminated waste or waste and fluids generated during surgery. Colour coding of different types of waste is used widely and it is important not to mix up waste that is dealt with in different ways. Waste or leftover fluids should not be put down the drain (which could result in splashes to the nurse), but should be handled as contaminated waste, in the prescribed manner. Waste bins and lids should be cleaned regularly.

## Cleaning

Remember to wash equipment between patients and again at the end of the day. You need to find out what the standards are for cleaning equipment and you must make sure that you and your colleagues clean everything at the beginning of a clinic, in between patients and at the end of the session.

Special items, such as curtains, still need to be cleaned, but not every day. Check your policy to see when special items need to be cleaned (i.e. monthly, quarterly, or bi-annually).

It is always worth asking yourself: ‘Would I want to be examined with this equipment? Am I confident I won't ‘catch’ anything from it?’ If the answer is no, then you really do need to make sure that you do something about it!

## Patient identification

Many countries place a waterproof wrist band onto a patient's arm containing the same identification information as in the patient notes. One can be checked against the other to ensure you are dealing with the correct patient. Colour coding can be used to indicate allergies and other conditions such as diabetes. A more affordable alternative is to write patient details on heavy-duty surgical tape applied to the surgical gown.

## Marking the surgical site

Marking the surgical site is also recommended to ensure surgery is conducted on the correct side of the body and to the correct body part. The surgeon may be responsible for marking the site, and in eye surgery, the mark is placed above the eye. Nursing staff must not rely on the fact that marking is always correct, because mistakes do happen. Nurses should check the chart and ask the patient too. Patients undergoing bilateral surgery should have a mark over both eyes.
